# Finding the optimal mix of smoking initiation and cessation interventions to reduce smoking prevalence

**DOI:** 10.1371/journal.pone.0212838

**Published:** 2019-03-01

**Authors:** Ruoyan Sun, David Mendez

**Affiliations:** Department of Health Management and Policy, University of Michigan, Ann Arbor, MI, United States of America; University of Wisconsin-Madison, UNITED STATES

## Abstract

There are more than one billion smokers globally according to the World Health Organization (WHO) report in 2017. Every year tobacco use causes nearly 6 million deaths worldwide. To deal with the smoking epidemic, society needs to invest resources efficiently. In this paper we introduce an optimal control model to determine the optimal mix of smoking initiation and cessation interventions to reduce smoking. We construct the model to reach a smoking prevalence target within a specific time horizon while minimizing cost. Our performance measure captures the cost of policy implementation over time, adjusting for inflation and social discounting. The analytical solutions to the model are presented in forms of ordinary differential equations (ODE). We then conduct several numerical simulations using data from the National Health Interview Survey (NHIS) and empirical studies. We first present analytical solutions for our model to solve for the optimal mix of smoking interventions. Then we simulate a public health policy to achieve 5% smoking prevalence in the US by 2030 using different combinations of real-life interventions. We examine the optimal trajectories, allocative efficiency and annual total cost of smoking cessation and initiation interventions. We find consistent results across all simulations. Our specific example reveals that the most efficient way to reach stated goal is by targeting cessation interventions first, and then gradually shifting resources to initiation interventions over time. While our numerical results are specific to the intervention we selected, our framework can be easily expanded to consider other potential interventions. We discuss the implications of our approach for the formulation of dynamic public health policies.

## Introduction

Currently, the average smoking prevalence globally is estimated to be 31.1% for men and 6.2% for women. That means approximately 967 million individuals worldwide smoke daily [1]. Tobacco use causes nearly 6 million deaths every year and smoking leads to many adverse health consequences [2]. For example, smoking increases the incidence of lung cancer, which is the leading cause of cancer death [3]. Studies have revealed that mortality among smokers was two or three times the mortality among non-smokers [4]. Despite successful tobacco policies in the past several decades, smoking has remained the leading preventable cause of disease and premature death in the US [5, 6]. The Surgeon General’s Report on smoking in 2014 estimated the annual smoking-attributable death to be around 480,000 [6].

In order to reduce the number of smokers in the US, policy makers have implemented two main categories of interventions: smoking prevention/initiation interventions and smoking cessation interventions. Smoking preventions focus on decreasing smoking initiation rate. The 2014 Surgeon General’s Report found that nearly 90% of adult smokers started before 18 and almost all by 26 [6]. As a result, initiation interventions usually target adolescents to prevent them from smoking. Some common smoking preventions are school-based programs and social media campaigns [7–10]. Smoking cessation, which encourages smokers to stop smoking, is another approach to reduce smoking [11–14]. Cessation programs can be divided into two groups: pharmacological interventions and non-pharmacological interventions. Pharmacological ones provide smokers prescribed drugs such as nicotine replacement treatment (NRT) to help them with addiction and withdrawal symptoms [15]. Non-pharmacological ones tend to take the forms of motivational interviews, counseling and behavioral therapy [16, 17]. Other policies such as smoke-free air laws and cigarette excise taxes have also contributed to the decrease in smoking prevalence [18].

One important outcome measure to evaluate these tobacco policies is smoking prevalence [19, 20]. Many countries and regions have incorporated smoking prevalence into their main policy goals. For example, researchers studied what the initiation and cessation rates need to be for New Zealand to achieve an under 5% smoking prevalence by 2025 [21]. However, policies implemented to reduce prevalence have not been derived following an optimization criterion to achieve their goals in the most efficient manner. To accelerate the eradication of this global smoking endemic, we need to find more efficient ways to decrease smoking prevalence by implementing smoking initiation and cessation interventions.

Previous studies have applied optimal control theory to help solve allocation problems in epidemiology and health care. For example, Area et al. studied how optimal vaccination schedule can help control the spread of Ebola [22]. Another study applies optimization method to search for optimal allocation of resources for nodding syndrome in Uganda [23]. Other popular applications of optimal control theory are in health care resources such as emergency department visits or Intensive Care Unit (ICU) usage [24, 25].

In this paper, we first establish a theoretical model to achieve policy goals using optimal control theory while minimizing cost. We consider the practical problem in which policy makers aim to achieve a certain smoking prevalence in a finite amount of time while minimizing the corresponding cost. This theoretical model is proposed and analyzed. We then conduct numerical simulations using the National Health Interview Survey (NHIS) data where the US population achieves a 5% smoking prevalence by 2030. Different combinations of empirical smoking initiation and cessation programs are used to investigate some general patterns. We also discuss the implications of this study for public health policies along with some limitations.

## Methods

### Model

We first introduce a dynamic equation describing the number of smokers in the population at time t. From previous work, we know $\dot{S}=\Phi \left(t\right)-\left[\mu \left(t\right)+\upsilon \left(t\right)\right]S\left(t\right)$ [26]. *S*(*t*) represents the number of smokers in the population at time t, and $\dot{S}$ denotes $\frac{dS(t)}{dt}$. Φ(*t*) is the number of new smokers at t, *μ*(*t*) the time variant death rate and *υ*(*t*) the time variant smoking cessation rate. We have *S*(*t*) and Φ(*t*) as number of people but *μ*(*t*) and *υ*(*t*) as rates. To facilitate interpretation of this model, we convert all parameters to rates. We assume a closed population of size *M*. Let *s*(*t*) be smoking prevalence in the population and *ϕ*(*t*) the smoking initiation rate, we can write this dynamic equation as: $\dot{s}=\phi \left(t\right)-\left[\mu \left(t\right)+\upsilon \left(t\right)\right]s\left(t\right)$ to describe smoking prevalence over time.

Next we construct our optimal control model with two types of interventions: smoking initiation and smoking cessation programs. Prevention programs aim to decrease the population initiation rate while the cessation programs attempt to increase the population cessation rate. We introduce the notion of units to describe the scale of interventions. For example, if we let 1 unit to be 1000 individuals, then 2 units of interventions imply a targeted population of 2000 people. The unit of initiation intervention is denoted by *u*_1_(*t*) while the cessation intervention by *u*_2_(*t*). In addition, we let *γ*_1_ to be the price per unit of initiation intervention and *γ*_2_ the unit price of cessation intervention, where *γ*_1_, *γ*_2_ > 0.

Because program costs are in nominal values, we need to adjust for inflation as well as real discount rate. We denote inflation rate as *i* and real discount rate as *r*. The inflation adjustment is incorporated into the model by multiplying the cost with (1 + *i*)^*t*^. Here we use exponential discounting and adjust inflation in the form of *e*^−*rt*^. As a result, the performance measure in our model adjusting for inflation and exponential discount rate is $\left(\gamma_{1}^{2}u_{1}^{2}\left(t\right)+\gamma_{2}^{2}u_{2}^{2}\left(t\right)\right){\left(1+i\right)}^{t}e^{-rt}$.

We apply four constraints that provide structure to the model. The first constraint is the dynamic equation of smoking prevalence introduced earlier. This equation describes how smoking prevalence changes over time as a function of birth rate, death rate and smoking cessation rate. Another constraint the model needs to follow is a linear estimation of smoking initiation rate in the population, aka *ϕ*(*t*). The population average smoking initiation rate changes as a result of the initial initiation rate and the effectiveness of prevention programs. *α*_0_ is the smoking initiation rate at initial time *t*_0_ and *α*_1_ is the effectiveness of the smoking prevention scaled to the population level. *α*_1_ < 0 since prevention programs decrease smoking initiation rate. We have *ϕ*(*t*) = *α*_0_ + *α*_1_ *u*_1_(*t*). The third constraint is related to the population smoking cessation rate, *θ*(*t*). Similarly, smoking cessation rate is determined by the initial cessation and death rate (*β*_0_), and the scaled effectiveness of smoking cessation programs (*β*_1_). We know that *θ*(*t*) = *β*_0_ + *β*_1_ *u*_2_(*t*). The last constraint is the final value of smoking prevalence. Since this is a fixed final time, fixed final state optimal control problem, the values of initial (*s*(*t*_0_)) and final smoking prevalence (*s*(*t*_*T*_)) are given as constants, so is the time period (*t*_*T*_−*t*_0_). We let constant *c* denote the final state of smoking prevalence, *s*(*t*_*T*_) = *c* where *c* > 0. We can use this optimal control model to solve for any tobacco control policy with a specific duration and smoking prevalence goal.

We are ready to formally introduce our model with the performance measure as well as four constraints.

$$\begin{array}{c} \begin{array}{cccc} & \underset{u_{1}\left(t\right),u_{2}\left(t\right)\geq 0}{\text{minimize}} & & \int_{t_{0}}^{t_{T}}\left(\gamma_{1}^{2}u_{1}^{2}\left(t\right)+\gamma_{2}^{2}u_{2}^{2}\left(t\right)\right){\left(1+i\right)}^{t}e^{-rt}dt\\ & \text{subject}\;\text{to}\; & & \dot{s}=\phi \left(t\right)-\theta \left(t\right)s\left(t\right)\\ & & & \phi \left(t\right)=\alpha_{0}+\alpha_{1}u_{1}\left(t\right)\\ & & & \theta \left(t\right)=\beta_{0}+\beta_{1}u_{2}\left(t\right)\\ & & & s(t_{T})=c\\ & \text{where} & & \gamma_{1},\gamma_{2},\beta_{1},c>0\\ & & & \alpha_{1}<0 \end{array} \end{array}$$ (1)

Using the Euler-Lagrange equation, we derive the following necessary conditions:

$$\{\begin{array}{l} 2\gamma_{1}^{2}u_{1}\left(t\right){\left(1+i\right)}^{t}e^{-rt}-\alpha_{1}p\left(t\right)=0\\ 2\gamma_{2}^{2}u_{2}\left(t\right){\left(1+i\right)}^{t}e^{-rt}+\beta_{1}p\left(t\right)s\left(t\right)=0\\ \alpha_{0}+\alpha_{1}u_{1}\left(t\right)=\left(\beta_{0}+\beta_{1}u_{2}\left(t\right)\right)s\left(t\right)+\dot{s}\\ p\left(t\right)\beta_{0}+p\left(t\right)\beta_{1}u_{2}\left(t\right)-\dot{p}=0 \end{array}$$ (2)

From the first two equations, we have $u_{1}\left(t\right)=\frac{\alpha_{1}p\left(t\right)e^{rt}}{2\gamma_{1}^{2}{\left(1+i\right)}^{t}}$ and $u_{2}\left(t\right)=\frac{-\beta_{1}p\left(t\right)s\left(t\right)e^{rt}}{2\gamma_{2}^{2}{\left(1+i\right)}^{t}}$. We plug these expressions into the last two equations to derive two time-variant ODEs as the necessary conditions. Appendix A provides more detailed technical descriptions of the model including the mathematical derivations.

### Numerical simulations

In this section, we conducted several policy simulations using our proposed model and empirical smoking interventions. We simulated three different combinations of tobacco policies. For each combination, there are two interventions: one smoking initiation intervention and one smoking cessation intervention. Our simulated tobacco control policy has two goals: 1) the smoking prevalence in the US reaches 5% by 2030; 2) minimizes the total implementation cost over this 13-year time horizon. To translate these scenarios into model language, we now have an optimal control problem with a fixed time horizon (*t*_0_ = 2017, *t*_*T*_ = 2030) and fixed final state (*s*_*T*_ = 5%).

We used the NHIS data in our model to simulate the US population for policy implementation. According to the NHIS data, the adult smoking prevalence in 2017 is 13.9% [27]. The annual population cessation, initiation, and death rates are taken to be 4.5%, 0.35%, and 0.89%, consistent with Mendez et al. Here initiation rate was defined as the 18-24 smoking prevalence taken as a proportion of the entire adult population. [26] We adopted these rates since they are the most recent population estimates. We set 2017 as our initial year and study the optimal intervention combination from 2017 to 2030 where the smoking prevalence reaches 5% in 2030. Using estimates of the adult population (249 million) and smoking prevalence (13.9%) in 2017, we estimated the total number of smokers as 34.7 million that year [27, 28]. We also used the annual national interest rate on the Federal Reserve website to calculate a five year average of inflation rate in the US between 2013 and 2017 [29]. The discount rate here is the conventional social discount rate at 3% [30]. Table 1 offers a summary of these model parameters.

**Table 1:** Demographic parameter values.

*α*_0_	*β*_0_	Inflation (i)	Discount (r)	*s*_0_	*s*_*T*_
0.0035	0.0539	0.0166	0.03	0.139	0.05

From previous work, we found two smoking initiation and three cessation programs meeting a specified search criterion. More detailed descriptions of these programs and criteria are in Appendix B. Because these interventions were carried out in different years, we discounted all costs to 2017 values using inflation data and exchange rates to 2017 US dollars. We also scaled up the programs’ effectiveness to generate population-level measures. The adjusted effectiveness and costs of these programs are shown in Table 2. In our theoretical model, we let *u*_1_(*t*) and *u*_2_(*t*) stand for units of interventions at each time *t*. Here we let one unit of intervention to be a million individuals. The size of a unit is arbitrary and does not affect our results. We adjusted the cost per unit of the program accordingly.

**Table 2:** Intervention parameter values.

Intervention	Type	Effectiveness (*α*_1_)	Effectiveness (*β*_1_)	Cost per million (2017 USD)
SCS	Cessation	-	0.095	$172,630,000
Tips	Cessation	-	0.065	$34,780,000
CLIQ	Cessation	-	0.00094	$216,867
Truth	Prevention	−3.59 × 10^−6^	-	$1,027,600
ASSIST	Prevention	−0.00011	-	$67,300,000

## Results

Out of the three smoking cessation and two prevention programs, we constructed three combinations of one cessation plus one prevention program based on different criteria. The first criterion we used is the highest effectiveness regardless of cost, and we chose A Stop Smoking In Schools Trial (ASSIST) and Smoking Cessation Services (SCS) [9, 13]. Then we picked ASSIST and Tips From Former Smokers (Tips), which are the most efficient interventions based on cost effectiveness ratio [7, 31]. Finally, we picked the last pair of Community Link to Quit (CLIQ) and the truth Campaign (truth) were picked based on the their lowest per unit cost [8, 12, 14, 32].

**Fig 1:**
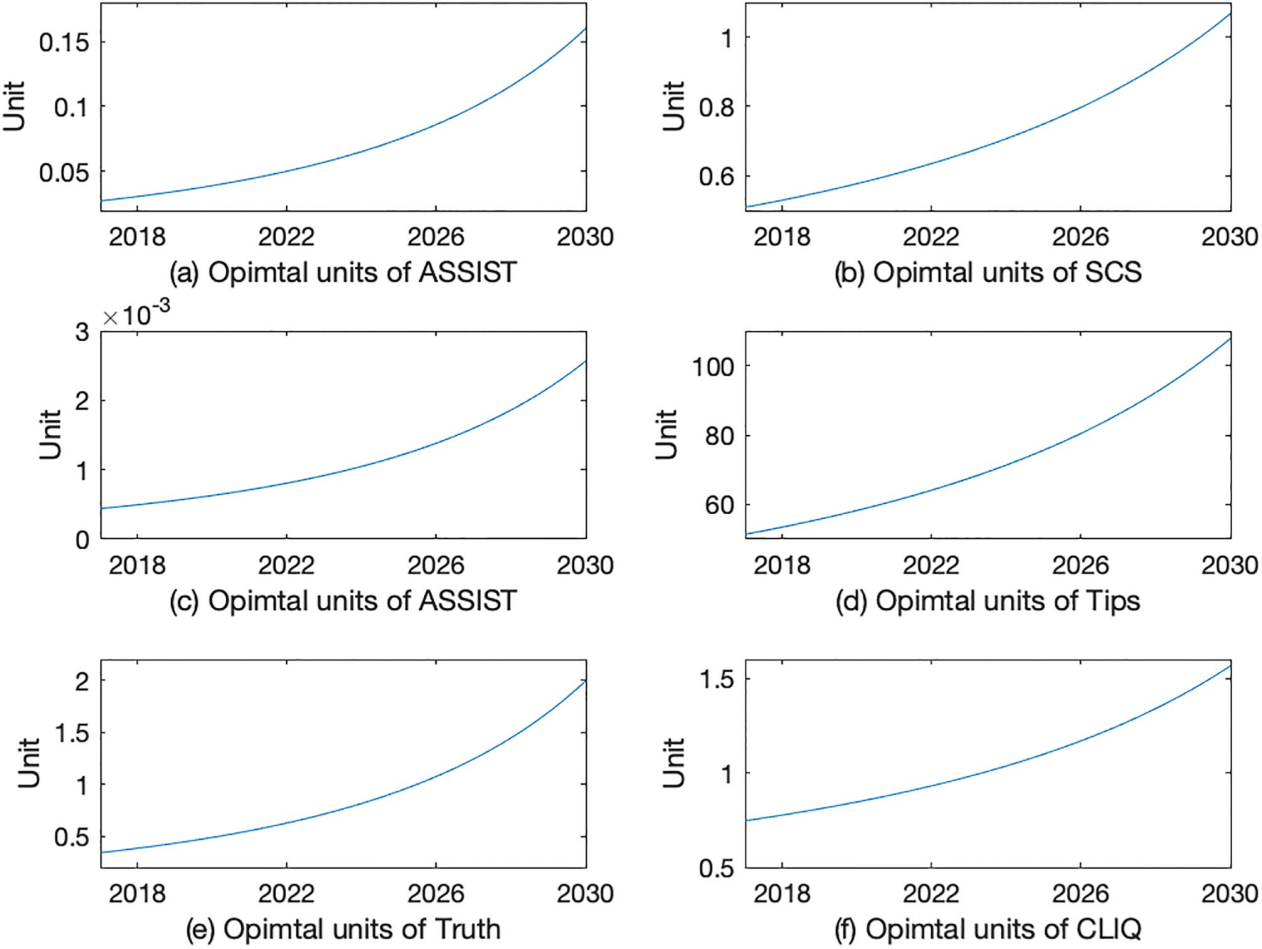
Optimal intervention units between 2017 and 2030.

Fig 1 has a total of six subgraphs describing the optimal trajectories of interventions. Each row represents one intervention combination. For example, 1a and 1b illustrate the optimal intervention trajectories for ASSIST and SCS that achieve the policy goal of reaching 5% smoking prevalence by 2030. The shapes of curves are consistent across three combinations. The curves for prevention programs increase monotonically and their increase accelerates over time. For ASSIST and SCS (AS), the prevention unit increases from 0.027 unit in 2017 to 0.16 in 2030. The prevention trajectory for ASSIST and Tips (AT) starts from 0.0004 unit in 2017 and grows to 0.0026 unit by 2030. In 2017, the prevention unit in truth and CLIQ (TC) is 0.34 unit. It then increases to 2 units in 2030. The cessation curves monotonically increase as well. The cessation program increases from 0.51 unit to 1.07 units for AS, 51.38 to 107.86 for AT and 0.75 to 1.55 for TC.

**Fig 2:**
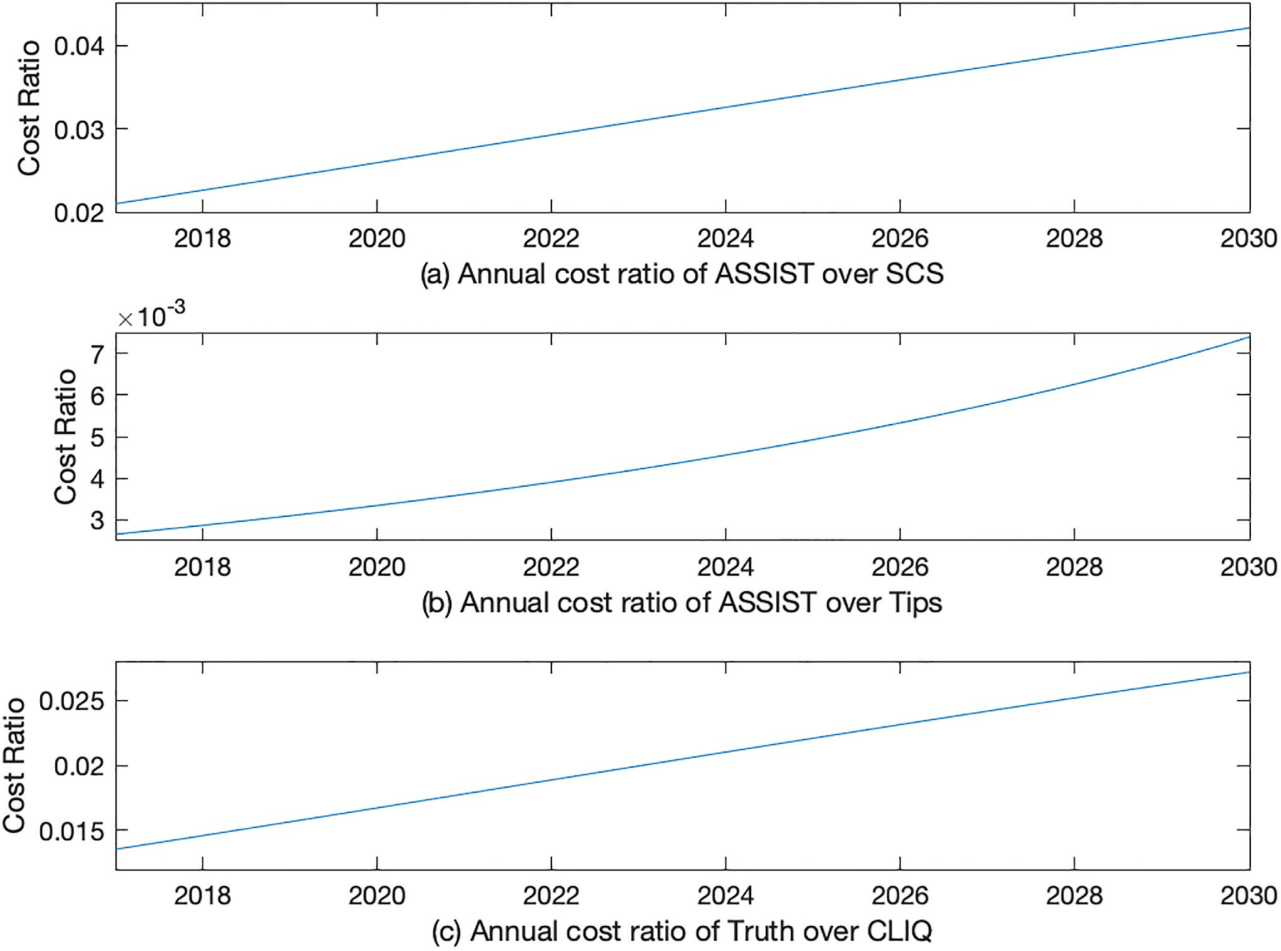
Optimal allocative efficiency: Cost of initiation over cessation.

Next we examined the optimal allocative efficiency between initiation and cessation programs. We calculated the nominal cost ratio of initiation over cessation for all three combinations. From Fig 2, we find that all cost ratios increase over time while their values are less than 1. The total cost ratio of initiation program over cessation program for AS increases from 0.021 to 0.042 over the 13-year span. The ratio grows from 0.0027 to 0.0074 for AT and from 0.014 to 0.027 for TC.

**Fig 3:**
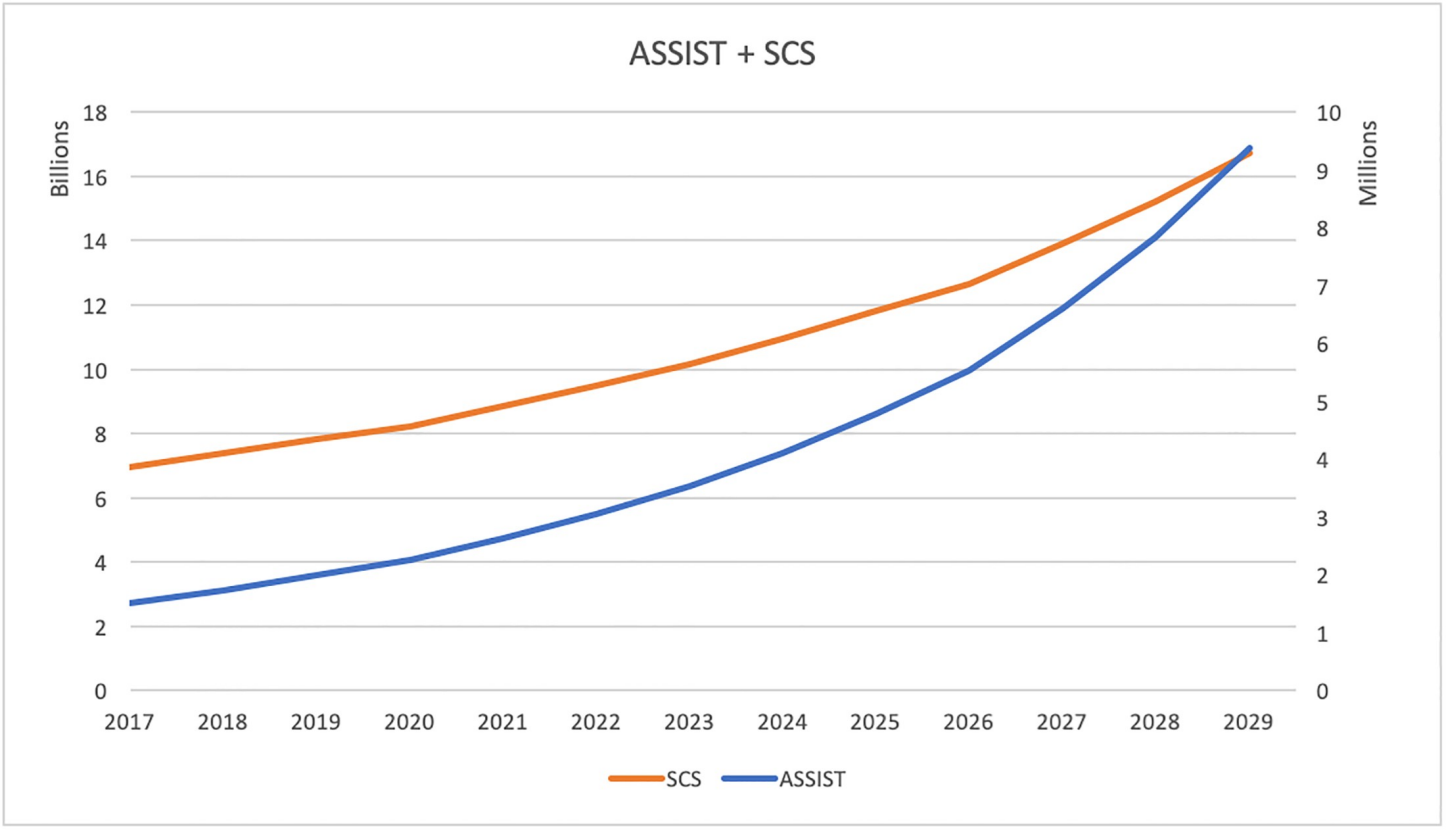
Estimated annual cost of ASSIST and SCS.

**Fig 4:**
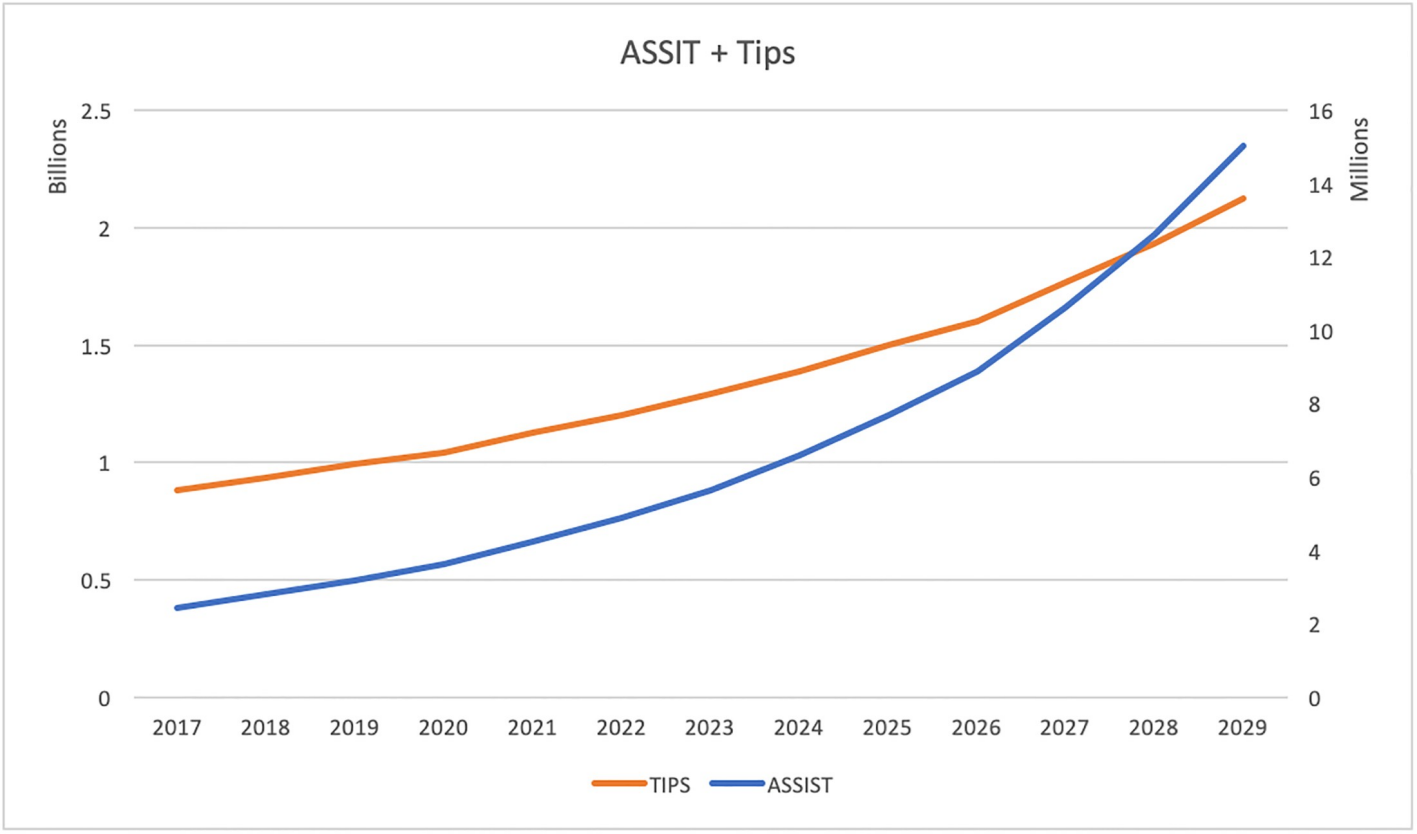
Estimated annual cost of ASSIST and Tips.

**Fig 5:**
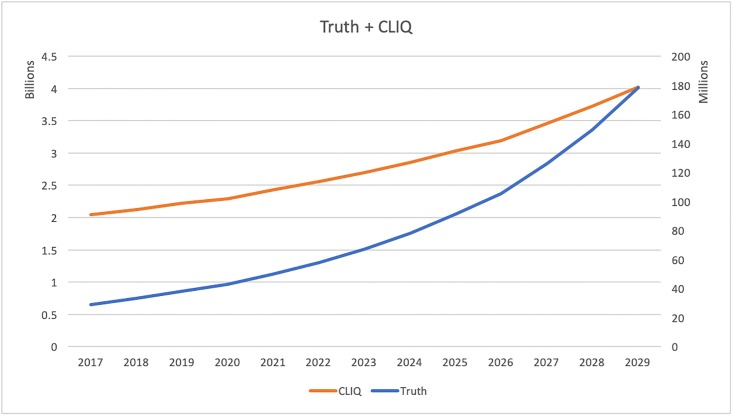
Estimated annual cost of truth and CLIQ.

Then we investigated the annual total costs of these interventions over time. We estimated the average annual cost for each intervention adjusting for inflation. Fig 3 plots the annual costs for AS, Fig 4 for AT, and Fig 5 for TC. The orange curves in these figures describe the cessation programs and use the y-axis to the left. The blue curves are costs for prevention and are based on the y-axis to the right. In Fig 3, the annual cessation cost goes up from 6.96 billion in 2017 to 16.74 in 2030. The prevention cost grows from 1.52 million to 9.38 million. Similarly in Fig 4, the annual cessation cost increases from 0.88 billion to 2.12 billion and prevention cost increases from 2.44 million to 15.05 million. Fig 5 shows the cessation goes up from 2.04 billion to 4.03 billion and prevention increases from 28.95 million to 178.5 million. The annual cessation cost goes up for all three policy simulations and the annual prevention cost grows over time as well.

## Discussion

We presented some numerical simulations to illustrate how this model can be applied to solve problems empirically. Based on our model, we know that if the status quo continues without any interventions, the predicted smoking prevalence in the US by 2030 will be 10.17%. We assume a public health policy of reducing smoking prevalence to 5% in the US by 2030 from 2017.

Trajectories of optimal interventions reveal the following trends: 1) the optimal trajectories for prevention programs increase monotonically and at a faster rate over time; 2) the cessation curves go up monotonically as well and accelerate over time. We find the patterns to hold across all three different intervention combinations of AS, AT and TC. The trajectories indicate a time-varying intervention mix is needed to achieve the policy goal in 13 years.

Then we examined the optimal allocative efficiency. Here, optimal allocative efficiency refers to the optimal distribution of resources between initiation and cessation efforts given a fixed budget. The allocative efficiency can also be considered as a cost trade-off between initiation and cessation programs. We discovered a consistent trend across three combinations: the ratio of nominal initiation cost over cessation cost increases over time but the value is always less than 1. The implication of these ratio curves has two parts. First, we should always invest more money in cessation programs, which makes sense because cessation intervention affects smoking prevalence immediately while initiation takes time to impact. But later we need to shift more focus to prevention programs over time. This shift is justified because when we have fewer smokers, the priority becomes preventing non-smokers from smoking. The dynamic perspective of this problem allows us to incorporate the differential effects of initiation and cessation efforts over time, which s a crucial contribution of this study.

Lastly, we studied how the annual cost of these programs change over 13 years. Based on Figs 3, 4 and 5, we found that the annual cost of cessation programs goes up and the cost of intervention programs increases over the years as well. This finding is consistent with our result of allocative efficiency, where more money should be invested to preventions over time. In this simulation example, if we want to eradicate smoking with a certain time horizon in mind, the most efficient way is first to target cessation interventions and then shift resources to initiation control. This example illustrates how our model can be applied to search for optimal mix of interventions empirically and how to interpret the findings.

The established theoretical model can be applied to many different situations to help policy makers adopt more efficient intervention combinations. Results from numerical simulations are case specific, depending on costs and effectiveness of policies as well as the underlying demography. For example, we can use this model to figure out the optimal cessation and prevention interventions in the UK when policy makers plan to achieve a 10% smoking prevalence in 5 years given appropriate parameter values.

This study has some limitations. First, results from some real-life studies could be biased. Some of those interventions carried out experiments on a subpopulation instead of a nationally representative sample. In addition, the ASSIST intervention was implemented in the UK. The demography between the US and UK could be very different. Another limitation is the assumption of constant intervention effectiveness. There could exist a diminishing return of interventions. That means it’s possible that we need to invest more resources to achieve the same effect as the smoking prevalence decreases. However, our model assumption is that we are expanding the programs to the population. The proportion of low hanging fruit should be the same across these groups. The assumption of linear effectiveness is reasonable. Despite these limitations, this study proposes a general framework to investigate the optimal path of interventions that meet certain policy goal while minimizing cost. Our model and simulations clearly reveals that there is a preferred sequence and emphasis of interventions.

## Conclusion

This study introduced a time-varying optimal control model to identify the optimal mix of initiation and cessation interventions to reduce smoking prevalence by a specific amount over a set time horizon at a minimum cost. By solving the optimal control model analytically, we derived the necessary conditions in terms of time-varying ordinary differential equations. We also obtained analytical solutions for the optimal intervention trajectories. This model addresses an important public health problem and attempts to fill in some gaps in the literature.

We conducted numerical simulations using US demographic data from NHIS together with real-life interventions to achieve a 5% smoking prevalence by 2030. Different combinations of smoking initiation and cessation interventions were adopted. We observed similar trends in development of optimal interventions, allocative efficiency and annual total cost of interventions across all combinations. Our simulation example revealed that the most efficient way to achieve policy goals is first to target cessation interventions and then shift resources to initiation interventions.

## Supporting information

S1 File Technical notes for the model. (PDF)

S2 File Empirical interventions. (PDF)
