# Supplementary material for: Finding the optimal mix of smoking initiation and cessation interventions to reduce smoking prevalence
Source: PLoS One. 2019 Mar 1;14(3):e0212838. doi: 10.1371/journal.pone.0212838 (PMC6396906; doi:10.1371/journal.pone.0212838)
Supplement: S1 File — (PDF) [file pone.0212838.s001.pdf]

S1. Technical notes for the model

$$\begin{aligned}
&\underset{u_1(t),u_2(t)\geq 0}{\text{minimize}} && \int_{t_0}^{t_T} (\gamma_1^2 u_1^2(t) + \gamma_2^2 u_2^2(t))(1+i)^t e^{-rt} dt \\
&\text{subject to} && \dot{s} = \phi(t) - \theta(t)s(t) \\
& && \phi(t) = \alpha_0 + \alpha_1 u_1(t) \\
& && \theta(t) = \beta_0 + \beta_1 u_2(t) \\
& && s(t_T) = c \\
&\text{where} && \gamma_1, \gamma_2, \beta_1, c > 0 \\
& && \alpha_1 < 0
\end{aligned} \tag{1}$$

We assume a closed population of size M and divide both sides of the equation by M. We obtain $\frac{\dot{S}}{M} = \frac{\Phi(t)}{M} - (\mu(t) + \upsilon(t))\frac{S(t)}{M}$. Let $s(t)$ be smoking prevalence in the population and $\phi(t)$ the smoking initiation rate, we can write this dynamic equation as: $\dot{s} = \phi(t) - [\mu(t) + \upsilon(t)]s(t)$ since $\dot{s} = \frac{d(\frac{S(t)}{M})}{dt} = \frac{1}{M}\frac{dS(t)}{dt} = \frac{1}{M}\dot{S}$.

We have four constraints in the model. The first one is the dynamic equation of smoking prevalence introduced earlier. The second is a linear estimation of smoking initiation rate $\phi(t)$. We let $\phi(t) = \alpha_0 + \alpha_1 u_1(t)$. α_0 is the smoking initiation rate at initial time t_0 and α_1 is the effectiveness of the smoking prevention scaled to the population level. Another way to think about α_1 is how effective per unit smoking prevention is at decreasing the population initiation rate, thus $\alpha_1 < 0$. The third constraint is the linear estimation of smoking cessation rate $\theta(t)$. Similarly, we can write $\theta(t) = \beta_0 + \beta_1 u_2(t)$. Here β_1 is the effectiveness of cessation interventions at the population level. β_1 measures how much the cessation program increases the average cessation rate in the population, thus $\beta_1 > 0$. β_0 has two parts: 1) the initial population average cessation rate at t_0, and 2) the death rate at t_0. We assume death rate to be constant, thus not changing over time. Since this is a fixed final time, fixed final state optimal control problem, the values of $s(t_0)$ and $s(t_T)$ are given as constants, so is the time period $t_T - t_0$. We let constant c denote the final state of smoking prevalence, $s(t_T) = c$ where $c > 0$. We can use this optimal control model to solve for any tobacco control policy with a specific duration and smoking prevalence goal.

We can combine the first three constraints into one by replacing $\phi(t)$ and $\theta(t)$ in the dynamic equation to get: $\dot{s} = \alpha_0 + \alpha_1 u_1(t) - (\beta_0 + \beta_1 u_2(t))s(t)$. The augmented integrand function is $g_a(s(t), u_1(t), u_2(t), p(t)) = (\gamma_1^2 u_1^2(t) + \gamma_2^2 u_2^2(t))(1+i)^t e^{-rt} + p(t)[\dot{s} - \alpha_0 - \alpha_1 u_1(t) + (\beta_0 + \beta_1 u_2(t))s]$. Using the Euler-Lagrange equation, we derive the following necessary conditions:

$$\begin{cases}
2\gamma_1^2 u_1(t)(1+i)^t e^{-rt} - \alpha_1 p(t) = 0 \\
2\gamma_2^2 u_2(t)(1+i)^t e^{-rt} + \beta_1 p(t)s(t) = 0 \\
\alpha_0 + \alpha_1 u_1(t) = (\beta_0 + \beta_1 u_2(t))s(t) + \dot{s} \\
p(t)\beta_0 + p(t)\beta_1 u_2(t) - \dot{p} = 0
\end{cases} \tag{2}$$

From the first two equations, we have $u_1(t) = \frac{\alpha_1 p(t) e^{rt}}{2\gamma_1^2 (1+i)^t}$ and $u_2(t) = \frac{-\beta_1 p(t) s(t) e^{rt}}{2\gamma_2^2 (1+i)^t}$. We plug these expressions into the last two equations to derive two time-variant ODEs as the necessary conditions.
