# Supplementary material for: Finding the optimal mix of smoking initiation and cessation interventions to reduce smoking prevalence
Source: PLoS One. 2019 Mar 1;14(3):e0212838. doi: 10.1371/journal.pone.0212838 (PMC6396906; doi:10.1371/journal.pone.0212838)
Supplement: S2 File — (PDF) [file pone.0212838.s002.pdf]

S2. Empirical interventions

In order to run numerical simulations of our model, we need information on both effectiveness of interventions as well as the implementation or societal cost of these interventions. We conducted a brief literature review to look for cost-effectiveness analysis of previous empirical smoking interventions. For smoking cessation programs, we used keywords ”smoking cessation” and ”cost-effectiveness” to search for randomized trials or randomized quasi-experiments carried out in the US in the past ten years in PubMed. We are able to find three qualified studies: 1. Smoking Cessation Services (SCS) ; 2. Tips from Former Smokers (Tips); and 3) Community Link to Quit (CLIQ). Due to a lack of randomized trials for smoking preventions, we extended the search range to 2000 and included one RCT in the UK: 1. the truth campaign; 2. A Stop Smoking in School Trials (ASSIST). Next we provide a brief summary fro each of these empirical interventions.

1. Smoking Cessation Services (SCS) [1]
A total of 223 smokers in California were randomized between 2006 and 2008 during psychiatric hospitalization and received stage-based smoking cessation services versus usual aftercare. The usual care group received a smoking cessation pamphlet and brief advice to quit during psychiatric hospitalization. The treatment group received a computer-assisted assessment of stage of change and subsequent interventions. A comparison between the treatment and control group yields a difference in abstinence rate of 18.75% versus 6.80 %, a relative risk of 2.76. In addition, the mean cost per person of smoking cessation services was $189 in treatment and $37 in usual care.

2. Tips From Former Smokers (Tips) [2,3]
The US Centers for Disease Control and Precention (CDC) launched a national, 3-month anttsmoking campaign in 2012 called Tips From Former Smokers (Tips). Tips featured emotional stories told by former smokers to increase awareness of the harmful health consequences of smoking. This campaign cost a total of 54 million dollars. During the campaign, 3051 smokers and 2220 non-smokers completed the baseline and follow-up assessments. Based on this sample, an analysis was conducted to yield national results. According to the study, an estimated 220,000 adult smokers become abstinent.

3. Community Link to Quit (CLIQ)(Proactive Tobacco Cessation Outreach to Smokers of Low Socioeconomic Status: A Randomized Clinical Trial) [4,5]
A total number of 707 low-SES adult smokers from 13 primary care practices affiliated with Partners HealthCare were recruited in the greater Boston area to this prospective, randomized clinical trial. This trial occurred between 2011 and 2013. Potential participants were identified through EHR records and 399 consented participants were randomized to the treatment group while 308 to the control group. Using the measurement of a self-reported past-7-day tobacco abstinence at 9 months after the randomization, the intervention group had a higher quit rate, 17.8%, compared to the usual care group, 8.1%. The total design and implementation of this intervention was estimated to be $283,023 for 8554 registry-identified smokers, resulting in a cost of $33.13 per identified smoker. We used a relative risk of 2.2 and per smoker cost of $33.13 in our model.

4. The truth campaign (truth) [6,7]
The truth campaign was launched between 2000 and 2002 by the American Legacy Foundation (ALF) to inform adolescents (12-17) of the deceptive practices within the tobacco industry and encourage them to make independent choices with regard to smoking. The campaign spent $324 million between 2000 and 2002 to develop, deliver, evaluate and litigate the truth campaign. By 2002, smoking rates among youth were 1.6% lower than they would have been without the campaign, approximately 300,000

fewer smokers as a result.

5. A Stop Smoking in Schools Trial (ASSIST) [8]

An intervention called A Stop Smoking In Schools Trial (ASSIST) was implemented in Year 8 students in the UK in 2001. A total of $10,730$ 12 to 13 year-old students from 59 schools in South East Wales and the West of England were included. Researchers conducted a cluster randomized controlled trial where influential students were identified to become peer supporters to encourage others not to smoke. Participants were followed up for 2 years in 29 control group schools and 30 treatment group schools. Using smoking prevalence at the 2-year follow-up as the major outcome measure, the authors found a significant 2.1% decrease in smoking prevalence in treatment groups. Furthermore, the authors examined the cost-effectiveness of ASSIST and estimated the cost of implementation to be £32 per student.

References

1. Barnett PG, Wong W, Jeffers A, Hall SM, Prochaska JJ (2015) Cost-effectiveness of smoking cessation treatment initiated during psychiatric hospitalization: Analysis from a randomized, controlled trial. J Clin Psychiatry 76(10):e1285-e1291.

2. McAfee T, Davis KC, Alexander RL, Pechacek TF, Bunnell R (2013) Effect of the first federally funded US antismoking national media campaign. Lancet 382(9909):2003-2011.

3. Xu X, Alexander RL, Simpson SA, Goates S, Nonnemaker JM, Davis KC, et al. (2015) A cost-effectiveness analysis of the first federally funded antismoking campaign. Am J Prev Med 48(3):318-25.

4. Haas JS, Linder JA, Park ER, Gonzalez I, Rigotti NA, Klinger EV, et al. (2015) Proactive tobacco cessation outreach to smokers of low socioeconomic status: a randomized clinical trial. JAMA Intern Med 175(2):218-26.

5. Levy DE, Klinger EV, Linder JA, Fleegler EW, Rigotti NA, Park ER, et al. (2016) Cost-effectiveness of a health system-based smoking cessation program. Nicotine Tob. Res. 00(00):1-8.

6. Holtgrave DR, Wunderink KA, Vallone DM, Healton CG (2009). Cost–Utility Analysis of the National truth® Campaign to Prevent Youth Smoking. Am J Prev Med 36(5):385-8.

7. Richardson AK, Green M, Xiao H, Sokol N, Vallone D (2010) Evidence for truth®: The young adult response to a youth-focused anti-smoking media campaign. Am J Prev Med 39(6):500-6.

8. Hollingworth W, Cohen D, Hawkins J, Hughes RA, Moore LA, Holliday JC, et al. (2011) Reducing smoking in adolescents: cost-effectiveness results from the cluster randomized ASSIST (A Stop Smoking In Schools Trial). Nicotine Tob. Res. 14(2):161-8.
